# Missense and Intronic Variants in *HNF1A* Affect Prostate Cancer Aggressiveness in Patients with Biochemical Recurrence

**DOI:** 10.7150/ijms.127638

**Published:** 2026-01-01

**Authors:** Min-Che Tung, Yung-Wei Lin, Chung-Howe Lai, Chia-Yen Lin, Lun-Ching Chang, Yu-Ching Wen, Shun-Fa Yang, Ming-Hsien Chien

**Affiliations:** 1 Division of Urology, Department of Surgery, Tungs' Taichung Metro Harbor Hospital, Taichung, Taiwan.; 2 Department of Life Science, College of Life Science, National Chung Hsing University, Taichung, Taiwan.; 3 Graduate Institute of Clinical Medicine, College of Medicine, Taipei Medical University, Taipei, Taiwan.; 4 International Master/PhD Program in Medicine, College of Medicine, Taipei Medical University, Taipei, Taiwan.; 5 Department of Urology, Wan Fang Hospital, Taipei Medical University, Taipei, Taiwan.; 6 Department of Urology, School of Medicine, College of Medicine and TMU Research Center of Urology and Kidney (TMU-RCUK), Taipei Medical University, Taipei, Taiwan.; 7 Division of Urology, Department of Surgery, Taichung Veterans General Hospital, Taichung, Taiwan.; 8 School of Medicine, Chung Shan Medical University, Taichung, Taiwan.; 9 School of Medicine, National Yang Ming Chiao Tung University, Taipei, Taiwan.; 10 Department of Mathematics and Statistics, Florida Atlantic University, Boca Raton, Florida, United States.; 11 Institute of Medicine, Chung Shan Medical University, Taichung, Taiwan.; 12 Department of Medical Research, Chung Shan Medical University Hospital, Taichung, Taiwan.; 13 Pulmonary Research Center, Wan Fang Hospital, Taipei Medical University, Taipei, Taiwan.; 14 Traditional Herbal Medicine Research Center, Taipei Medical University Hospital, Taipei, Taiwan.; 15 TMU Research Center of Cancer Translational Medicine, Taipei Medical University, Taipei, Taiwan.; 16 School of Medical Laboratory Science and Biotechnology and Ph.D. Program in Medical Biotechnology, College of Medical Science and Technology, Taipei Medical University, Taipei, Taiwan.

**Keywords:** prostate cancer, hepatocyte nuclear factor 1 alpha, single-nucleotide polymorphism, biochemical recurrence, clinicopathologic progression

## Abstract

Prostate cancer (PCa) is a genetically and phenotypically heterogeneous disease, and further advancements in PCa biomarker discovery are urgently required. Hepatocyte nuclear factor 1 A (HNF1A), a transcription factor, plays a critical role in PCa progression after biochemical recurrence (BCR). However, studies investigating the impact of *HNF1A* genetic variants on PCa are scarce. Therefore, in this study, we explored the associations of *HNF1A* single-nucleotide polymorphisms (SNPs) with susceptibility to BCR in PCa and its clinicopathological development. Two nonsynonymous (missense) SNPs [rs2464196 (S487N) and rs1169288 (I27L)] and two intronic SNPs [rs1169286 and rs735396] were analyzed using a TaqMan allelic discrimination assay for genotyping in a cohort of 690 Taiwanese patients with PCa. The results demonstrated that patients with PCa carrying the *HNF1A* rs735396 (TC+CC), rs2464196 (GA+AA), or rs1169288 (AC+CC) had a higher risk of developing tumors with higher pathological Gleason grades (3-5). These associations were particularly evident in the BCR subpopulation. Moreover, analysis of data from The Cancer Genome Atlas revealed that *HNF1A* expression was higher in PCa tissues than in normal tissues. Moreover, higher *HNF1A* expression was correlated with higher Gleason scores, more advanced pathological T stages, and metastasis. Taken together, our findings indicated that elevated HNF1A expression promotes PCa progression and that the missense SNPs rs2464196 and rs1169288, as well as the intronic SNP rs735396, may influence *HNF1A* expression, thereby influencing PCa aggressiveness, particularly in patients with BCR.

## Introduction

Prostate cancer (PCa) is estimated to account for 288,300 new cases in the United States; it is the most frequently diagnosed cancer and the second leading projected cause of cancer-related death among men 1. Most patients with PCa are identified through early-stage prostate-specific antigen (PSA) screening, and early-stage PCa is typically treatable with radical prostatectomy (RP) 2. However, serum PSA levels can increase after RP, leading to biochemical recurrence (BCR) of PCa and increasing the risks of metastasis and death 2, 3. Some PCa cases progress rapidly after BCR and transform into an aggressive type of disease called castration-resistant PCa (CRPC). Many patients die of CRPC within 2 years of diagnosis 4. Consequently, progression to CRPC can be a powerful surrogate marker for PCa prognosis, even in patients undergoing RP and subsequently developing BCR. Several common markers, such as Gleason score, PSA kinetics, lymphovascular invasion, and T stage, have been reported to predict post-RP progression from BCR to CRPC 5, 6. In addition to these markers, several novel markers are currently under investigation.

Hepatocyte nuclear factor 1A (HNF1A), an HNF1 family protein initially identified in the liver, has been confirmed to be expressed in several organs. *HNF1A*, located on human chromosome 12q24.3, encodes a transcription factor containing a homeodomain 7. HNF1A has been noted to have oncogenic roles in various cancers. For example, HNF1A was noted to promote pancreatic cancer stem cell growth 8. Moreover, elevated HNF1A expression was reported to enhance radiation resistance via PI3K/AKT pathway activation in esophageal squamous cell carcinoma cells 9. Furthermore, upregulation of both matrix metalloproteinase 14 (MMP14) and HNF1A was reported to promote cell motility and metastasis through induction of epithelial-mesenchymal transition (EMT) in cervical cancer cells 10. However, HNF1A has also been noted to have tumor-suppressive roles in certain contexts. For example, HNF1A was reported to increase chemosensitivity to gemcitabine by targeting ABCB1 in pancreatic cancer cells 11. Furthermore, the combined expression of HNF1A, HNF4A, and forkhead box protein A3 (FOXA3) was noted to inhibit hepatocellular carcinoma cell growth 12.

HNF1A is highly expressed in PCa cells, and its knockdown can suppress tumor growth 13. Aberrant HNF1A expression was reported to be associated with abbreviated responses to androgen deprivation therapy (ADT) in patients with BCR and progression to CRPC 13. Moreover, HNF1A is a critical driver of taxane resistance in CRPC 14. Consequently, targeting HNF1A using bromodomain and extraterminal domain inhibitors was proposed as a therapeutic intervention for CRPC 15. Although several studies have investigated the functional role of HNF1A in PCa progression, the effects of *HNF1A* genetic variants on PCa remain unexplored. Missense mutations represent the predominant mutation type of *HNF1A* in various cancers, including PCa 16. In the current study, we examined the associations of missense and intronic single-nucleotide polymorphisms (SNPs) within *HNF1A* with the risk of BCR and clinicopathological development of PCa in a Taiwanese population.

## Materials and Methods

### Study populations and ethics

We collected whole-blood samples from 690 patients with PCa who underwent robotic-assisted laparoscopic RP at Taichung Veterans General Hospital (Taichung, Taiwan) between 2012 and 2018. Written informed consent was obtained from all participants before venous blood collection, and the study protocol was approved by the Institutional Review Board of Taichung Veterans General Hospital (IRB no. CE19062A-2). Clinical data at diagnosis, including pathologic Gleason score, clinical and pathologic T and N stages, seminal vesicle invasion status, perineural invasion status, lymphovascular invasion status, and D'Amico risk classification, were retrieved from the patients' medical records. BCR in the recruited PCa patients was defined as the detection of two consecutive PSA measurements, each exceeding 0.2 ng/mL. This threshold served as an indicator of potential cancer recurrence following initial treatment. In addition, the interval between the two PSA measurements was confirmed to rule out transient fluctuations, ensuring that the elevation represented a true biochemical relapse. This definition is consistent with widely accepted clinical guidelines for post-treatment monitoring in PCa.

### Genomic DNA extraction

Whole-blood samples were collected in EDTA-containing tubes and centrifuged. Next, their buffy coats were isolated, and genomic DNA was extracted using a QIAamp DNA Blood Mini Kit (Qiagen, Valencia, CA, USA) in accordance with the manufacturer's instructions. The quality of the extracted DNA was evaluated on a Nanodrop-2000 spectrophotometer (Thermo Scientific, Waltham, MA, USA). Subsequently, high-quality extracted DNA was used as the template for polymerase chain reaction (PCR).

### Selection and determination of *HNF1A* genetic polymorphisms

Four SNPs in *HNF1A* were selected for analysis, including two missense variants [rs2464196 (G/A) and rs1169288 (A/C)] and two intronic variants [rs735396 (T/C) and rs1169286 (T/C)]. These SNPs were selected because they may be associated with different cancer types 17-19 or other diseases (e.g., metabolic syndrome and coronary artery disease) 20-22. We conducted genotyped rs2464196 (assay ID: C___1263617_10), rs1169288 (assay ID: C___7474231_10), rs735396 (assay ID: C___1263608_1_), and rs1169286 (assay ID: C___1263544_20) assays by using TaqMan SNP Genotyping Assay on an ABI StepOnePlus Real-Time PCR System (Thermo Fisher Scientific). The detailed procedures for DNA genotyping were described in our previous study 23.

### Bioinformatics analysis

RNA expression data and the corresponding clinical information of The Cancer Genome Atlas (TCGA)-prostate adenocarcinoma (PRAD) cohort were obtained from the UCSC Xena database (https://xena.ucsc.edu/) (dataset ID: TCGA-PRAD.star_tpm.tsv). The database provides gene expression data in the log2(TPM+1) format; thus, we converted these data back to the TPM (i.e., transcripts per million) format for subsequent analyses. Clinical variables, including Gleason scores and TNM stages, were extracted, and all data were organized according to the corresponding TCGA IDs. Differences between two independent groups were assessed using an unpaired Student's *t* test, whereas a paired Student's *t* test was applied for groups with NT-paired samples. For comparisons across multiple groups, we performed one-way analysis of variance followed by Tukey's post hoc test. Spearman correlation analysis was used to identify genes correlated with *HNF1A*. These genes were then ranked according to their correlation coefficients and subjected to gene set enrichment analysis (GSEA). Pathways with a false discovery rate (FDR) < 0.05 were considered statistically significant. Overall survival (OS) was assessed by categorizing patients with PRAD into high- and low-*HNF1A* expression groups according to the best cutoff values, and statistical significance was determined using the log-rank test.

### Statistical analysis

Between-group differences in demographic characteristics were analyzed using the chi-square and Student's *t* tests. Associations between genotypic frequencies and clinicopathological features were examined using multivariate logistic regression models, with these yielding odds ratios (ORs) and adjusted ORs (AORs) with their respective 95% confidence intervals (CIs). All statistical analyses were performed using SAS (version 9.1, 2005, for Windows; SAS Institute, Cary, NC, USA). Significance was defined on the basis of *p* < 0.05.

## Results

### Demographic characteristics of enrolled patients with PCa

Table 1 presents a comparison of the demographic and clinical characteristics of patients with PCa with postoperative BCR (n = 219) and those without (n = 471). The patients with BCR were more likely to present with advanced clinical T stages (T3-T4) at diagnosis. Furthermore, according to pathological assessment results, the BCR cases more frequently involved higher Gleason grades (3-5) and advanced pathologic T (T3-T4) and N (N1) stages, along with seminal vesicle, perineural, and lymphovascular invasion. According to the D'Amico risk classification, more patients with BCR were classified as being at high risk. In general, the demographic and clinical profiles of our PCa cohort, regardless of BCR status, were comparable to those reported previously 24.

### Potential impact of *HNF1A* genetic variants on postoperative BCR in patients with PCa

We next examined the potential influence of four selected *HNF1A* SNPs [i.e., rs735396 (T/C), rs2464196 (G/A), rs1169288 (A/C), and rs1169286 (T/C)] on postoperative BCR in patients with PCa undergoing RP. The genotype distributions of these SNPs were first evaluated in our cohort of 690 patients, with the most frequent variants being heterozygous T/C, G/A, A/C, and T/C, respectively (Table 2). AORs with 95% CIs were estimated using multivariate logistic regression models controlled for potential confounders to assess the associations between *HNF1A* SNPs and BCR risk. The analyses revealed no significant associations between these SNPs and postoperative BCR; these results were consistent across both dominant and codominant genetic models (Table 2).

### Relationships between clinicopathological features and *HNF1A* genetic variants in patients with PCa

We further investigated the influence of *HNF1A* SNPs on the clinicopathological characteristics of PCa, including pathologic T and N stages, Gleason grades, clinical T stage, tumor invasion, and D'Amico risk classification. Regarding the four analyzed *HNF1A* SNPs, patients with PCa harboring at least one minor allele of rs735396 (TC+CC), rs2464196 (GA+AA), or rs1169288 (AC+CC) demonstrated a significant increase in risk of tumors with higher Gleason grades (3, 4, or 5) compared with those with wildtype homozygotes (ORs = 1.672, 1.635, and 1.399, respectively; Tables 3 and 4). By contrast, rs1169286 was not significantly associated with pathological Gleason grades (Table 4).

### Significant correlation of pathological Gleason grades with *HNF1A* SNPs rs735396 and rs2464196 in PCa patients with BCR

Given the critical role of HNF1A in BCR 13, we stratified patients with PCa into BCR and non-BCR subgroups and assessed the associations between *HNF1A* SNPs and clinicopathological features in each subgroup. Notably, of the patients with BCR, rs735396 (TC+CC) and rs2464196 (GA+AA) carriers demonstrated significantly higher risks of tumors developing higher Gleason grades (3, 4, or 5) compared with the overall PCa population [OR = 2.628 (95% CI = 1.377-5.017, *p* = 0.003) and OR = 2.401 (95% CI = 1.268-4.547, *p* = 0.006), respectively; Tables 5 and 6]. By contrast, these associations were not observed in patients without BCR.

### Correlation of elevated *HNF1A* expression in PCa tissues with higher Gleason scores, larger tumor size, and distant metastasis

**Figure 1:**
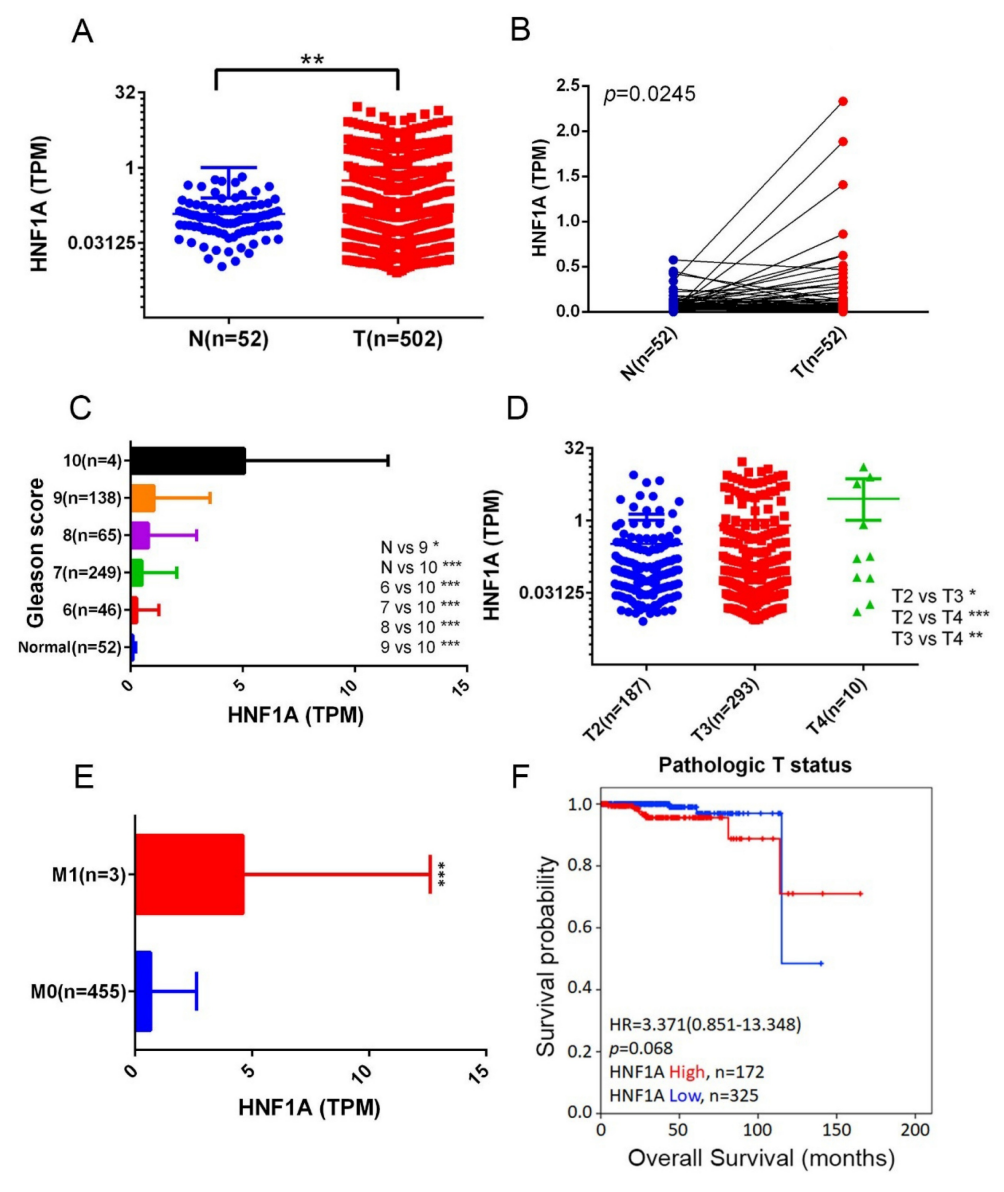
Correlation between *HNF1A* expression and clinical features in patients with PCa based on TCGA-PRAD data. (A) *HNF1A* expression levels in unpaired normal and tumor tissues in the TCGA-PRAD dataset are presented. (B) *HNF1A* expression levels were analyzed in 52 matched PCa tissues and their corresponding normal tissues. (C-E) *HNF1A* expression levels in PCa from TCGA-PRAD were compared based on the Gleason scores (C), pathological T stages (D), and distant metastasis (E). (F) Kaplan-Meier survival curves were used to illustrate OS of patients with high and low *HNF1A* expression.

We next analyzed HNF1A expression levels in normal and PCa tissues and examined their correlations with PCa progression and prognosis by using data from the TCGA-PRAD dataset. The results demonstrated that *HNF1A* expression was significantly higher in PCa tissues than in noncancerous tissues (Figure 1A) and their corresponding matched normal counterparts (Figure 1B). Moreover, relative *HNF1A* transcript levels were elevated in patients with PCa with higher Gleason scores (Figure 1C), advanced T stages (Figure 1D), and distant metastasis (Figure 1E). A Kaplan-Meier analysis further indicated that higher *HNF1A* expression levels tended to be associated with shorter OS times (Figure 1F).

### Potential HNF1A-regulated molecular mechanisms underlying PCa progression

**Figure 2:**
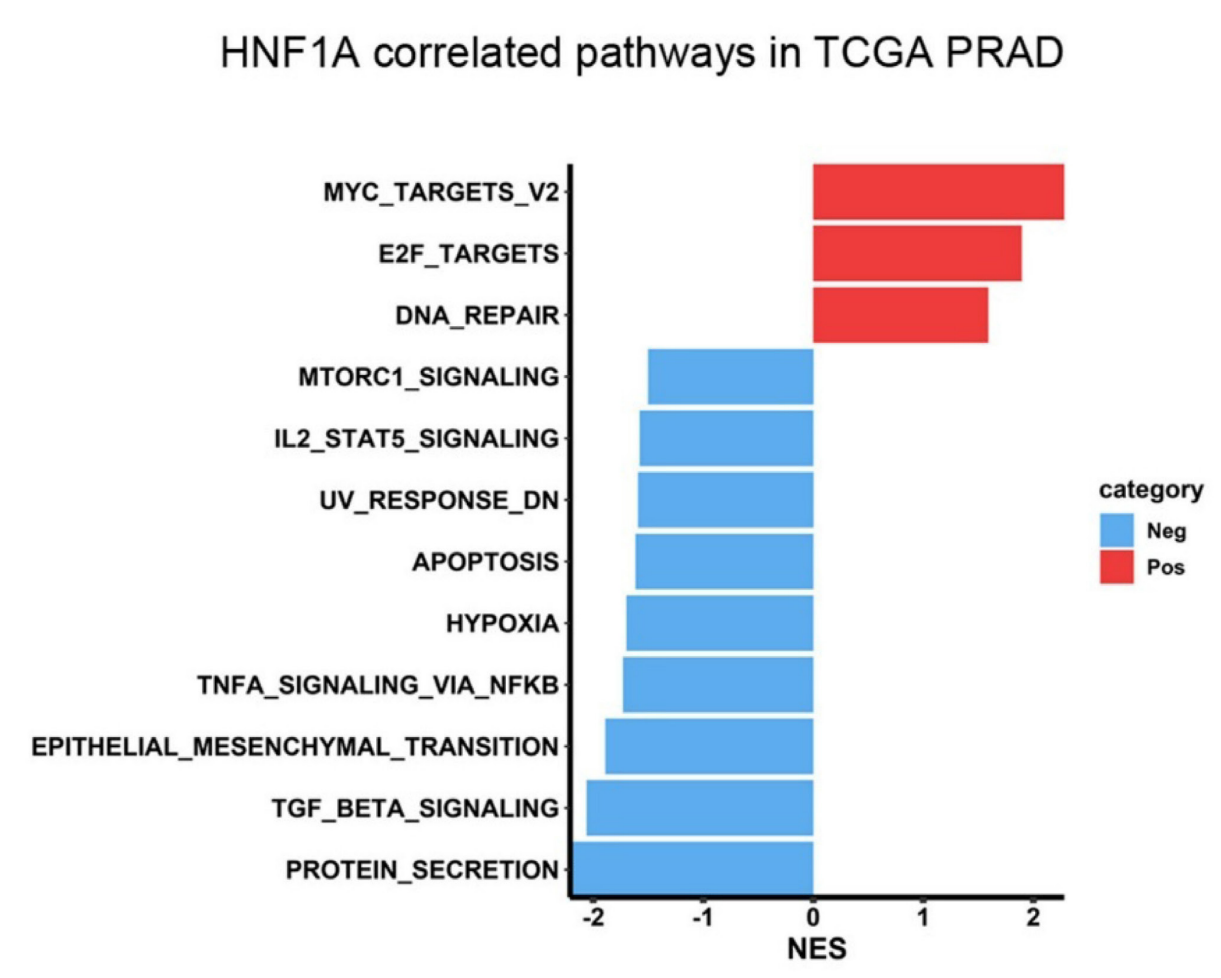
HNF1A-associated pathways in patients with PCa. The horizontal bar plot displays pathways identified in the Hallmark database that are correlated with *HNF1A* expression. Pathways positively and negatively associated with *HNF1A* are displayed in red and blue, respectively. The *x*-axis indicates normalized enrichment scores (NESs).

To investigate the mechanisms through which HNF1A contributes to PCa progression, we performed GSEA by using data from the TCGA-PRAD dataset. The results demonstrated that “MYC targets” and “E2F targets” were the top two Hallmark gene sets enriched in the HNF1A-high group (Figure 2). Notably, MYC is a major driver of PCa tumorigenesis and progression; elevated MYC expression can accelerate PCa development, increasing Gleason scores and promoting metastasis, BCR, and CRPC development 25, 26. Moreover, higher E2F expression is associated with higher Gleason scores, advanced tumor stage, metastasis, and elevated BCR risk 27. Therefore, our results indicated that HNF1A may promote PCa progression by regulating MYC- and E2F-related pathways.

## Discussion

PCa is generally treatable when detected early and monitored closely. However, advanced-stage PCa tumors frequently acquire resistance to ADT, which results in progression to CRPC, a lethal form of PCa 28, 29. Given the oncogenic role of the transcription factor HNF1A in PCa progression 13-15, we examined missense and intronic SNPs within *HNF1A* in relation to BCR. Distinct SNP distributions were observed between patients with and without BCR. In particular, carriers of the mutant C allele of rs735396 and rs1169288 or the mutant A allele of rs2464196 exhibited a significantly elevated risk of PCa tumors with higher Gleason grades under a dominant model (TC+CC, AC+CC, or GA+AA). These associations, particularly for rs735396 and rs2464196, were more pronounced in patients with BCR than in those without. In addition, the analysis of PCa tissue samples revealed that elevated *HNF1A* expression was significantly correlated with higher pathological Gleason scores, larger tumor sizes, and tumor metastasis, as well as enrichment of pathways related to MYC and E2F targets. Taken together, these findings suggest that *HNF1A* genetic variants and expression may jointly contribute to PCa progression and aggressiveness.

Inflammation has been increasingly recognized as a critical factor in the pathogenesis and progression of many solid tumors, including PCa 30. C-reactive protein (CRP), an inflammation marker and acute-phase protein produced in response to inflammation, is correlated with tumor progression and prognosis in several cancers, including PCa 31-33. Patients with PCa with adverse pathological features (e.g., high Gleason scores, extracapsular extension, seminal vesicle invasion, lymph node metastasis, and positive surgical margins) demonstrate elevated preoperative CRP levels. Furthermore, patients with PCa with elevated CRP levels have significantly lower 5-year BCR-free survival rates than do those with normal CRP levels 34.

HNF1A, mainly expressed in the liver, acts as a transcription factor. CRP is also primarily synthesized in the liver by hepatocytes 35. Thus, HNF1A may serve as a crucial regulator of CRP production. Contemporary evidence has confirmed that HNF1A binds to the promoter region of *CRP*, thereby upregulating *CRP* expression and promoting laryngeal cancer progression 36. Moreover, genome-wide association studies have identified several *HNF1A* genetic variants, including rs735396, rs1169288, and rs2464196, that are associated with circulating CRP levels 37, 38. Taken together, these findings suggest that rs735396, rs1169288, and rs2464196 regulate *HNF1A* expression, thereby influencing CRP levels and ultimately affecting surgical Gleason scores. Similar to those with PCa, patients with hepatocellular carcinoma (HCC) harboring the AA genotype of rs2464196 demonstrate significantly elevated AFP, AST, and ALT levels, which promote HCC progression 17, 18.

rs1169288 and rs2464196 are nonsynonymous coding variants, also called missense SNPs, located in the exonic regions of *HNF1A*. A missense SNP can alter the amino acid sequence of the encoded protein, potentially affecting the protein's stability and its ability to interact with other molecules 39, 40. Thus, we hypothesize that rs1169288 and rs2464196 influence the structure of HNF1A, thereby enhancing its binding to the *CRP* promoter, upregulating *CRP* expression, and ultimately exacerbating PCa aggressiveness; this hypothesis requires validation in future studies. In contrast to rs1169288 and rs2464196, rs735396 is located within intron 9 of *HNF1A*. Polymorphisms in intronic regions do not typically alter the protein sequence. Nevertheless, emerging evidence indicates that these types of variations may influence cancer susceptibility through both genetic and epigenetic mechanisms. Intronic sequences often harbor cis-acting regulatory elements, such as transcription factor binding sites, enhancers, and silencers, all of which can positively or negatively regulate gene expression 41. Jiang et al. proposed that rs735396 might reside within an enhancer element, where this SNP could affect interactions with DNA-binding factors and thereby regulate *HNF1A* expression. They further demonstrated that the T allele may reduce *HNF1A* expression by altering enhancer activity in HCC cells 42. Nevertheless, the impact of rs735396 on *HNF1A* expression in PCa cells remains to be elucidated in future studies.

It is well documented that PCa tumor foci exhibit marked overexpression of *MYC* mRNA and protein, which correlates with increased disease severity, including higher Gleason scores, BCR, and metastasis 43, 44. Moreover, MYC overexpression in normal luminal cells of the murine prostate is sufficient to initiate PCa 45. Taken together, these findings demonstrate that *MYC* is an oncogene that is a substantial driver of tumorigenesis and progression in PCa. In the current study, we noted that *HNF1A* expression was positively associated with MYC target gene-related signatures in the TCGA-PRAD dataset. Several studies have reported that MYC transcriptionally activates the long noncoding RNA *HNF1A-AS1*, promoting progression in various cancers 46, 47. HNF1A can activate *HNF1A-AS1* transcription by directly binding to its promoter region in HCC 48. Therefore, HNF1A may cooperate with MYC to promote PCa progression by regulating *HNF1A-AS1*; however, this hypothesis requires further investigation. E2F transcription factors are implicated in PCa because they are strongly expressed in tumors and promote cancer cell growth by regulating the cell cycle and other cellular processes. Higher expression of specific E2F members (E2F1-3) is associated with more advanced tumors, higher Gleason scores, and increased posttreatment BCR risk 27. We also observed that *HNF1A* expression was associated with E2F target gene-related signatures in the TCGA-PRAD dataset. Research indicates that MYC induces the transcription of *E2F1-3* genes 49, suggesting that MYC-regulated E2F expression is involved in HNF1A-mediated PCa progression. However, the interactions among HNF1A, MYC, and E2F in PCa progression warrant further investigation.

In summary, this is the first study to investigate the distinct allelic effects of both missense and intronic *HNF1A* SNPs in a Taiwanese population, highlighting their potential roles in PCa progression. Clinically, we identified an oncogenic role of *HNF1A* in PCa specimens. Moreover, our findings indicate that HNF1A-related signaling pathways, including MYC and E2F targets, may be key drivers of PCa progression. SNP profiling of noninvasive biopsies to predict cancer risk and disease progression can provide valuable insights for precision medicine. In particular, the missense SNPs rs2464196 (S487N) and rs1169288 (I27L), along with the intronic SNP rs735396, may serve as critical markers of PCa aggressiveness, particularly in patients with BCR.

However, our study still has several limitations that should be acknowledged. First, all PCa patients included in the SNP analysis were Taiwanese (of Asian ethnicity), whereas the correlations between *HNF1A* expression and clinicopathologic features or prognosis were assessed using the TCGA-PRAD dataset, which is composed predominantly of Caucasian and African American individuals. Therefore, additional studies are required to validate the associations between HNF1A expression and clinicopathologic characteristics specifically in Taiwanese PCa tissues. Second, future investigations should simultaneously collect both mRNA and DNA from the same PCa patient samples to better assess the impact of *HNF1A* SNPs on gene expression. Finally, whether the missense SNPs rs2464196 and rs1169288, as well as the intronic SNP rs735396, can serve as reliable markers for predicting PCa aggressiveness should be further examined across diverse racial and ethnic populations.

## Figures and Tables

**Table 1:** Distributions of demographic characteristics of included patients.

Variable	BCR		
	No (*n* = 471)	Yes (*n* = 219)	*p*
Age at diagnosis (years)			
≤65	199 (42.3%)	90 (41.1%)	0.775
>65	272 (57.7%)	129 (58.9%)	
Pathologic Gleason grade group			
1 or 2	343 (72.8%)	70 (32.0%)	0.001*
3, 4, or 5	128 (27.2%)	149 (68.0%)	
Clinical T stage			
1 or 2	429 (91.1%)	164 (74.9%)	0.001*
3 or 4	42 (8.9%)	55 (25.1%)	
Clinical N stage			
N0	464 (98.5%)	212 (96.8%)	0.138
N1	7 (1.5%)	7 (3.2%)	
Pathologic T stage			
2	311 (66.0%)	52 (23.7%)	0.001*
3 or 4	160 (34.0%)	167 (76.3%)	
Pathologic N stage			
N0	459 (97.5%)	172 (78.5%)	<0.001*
N1	12 (2.5%)	47 (21.5%)	
Seminal vesicle invasion			
No	426 (90.4%)	117 (53.4%)	<0.001*
Yes	45 (9.6%)	102 (46.6%)	
Perineural invasion			
No	163 (34.6%)	18 (8.2%)	<0.001*
Yes	308 (65.4%)	201 (91.8%)	
Lymphovascular invasion			
No	437 (92.8%)	142 (64.8%)	<0.001*
Yes	34 (7.2%)	77 (35.2%)	
D'Amico risk classification			
Low or Intermediate risk	266 (56.5%)	75 (34.2%)	<0.001*
High risk	205 (43.5%)	144 (65.8%)	

* *p* value < 0.05 as statistically significant.

**Table 2:** Distribution frequencies of *HNF1A* genotypes in included patients.

Variable	BCR			
	No (*n* = 471)	Yes (*n* = 219)	aOR (95% CI)	*p*
rs735396				
TT	122 (25.9%)	51 (23.3%)	1.000 (reference)	
TC	219 (46.5%)	118 (53.9%)	0.933 (0.585-1.487)	0.771
CC	130 (27.6%)	50 (22.8%)	0.747 (0.431-1.294)	0.298
TC+CC	349 (74.1%)	168 (67.7%)	0.868 (0.559-1.348)	0.528
rs2464196				
GG	124 (26.3%)	53 (24.2%)	1.000 (reference)	
GA	220 (46.7%)	116 (53.0%)	0.901 (0.568-1.431)	0.660
AA	127 (27.0%)	50 (22.8%)	0.749 (0.433-1.294)	0.300
GA+AA	347 (73.7%)	166 (75.8%)	0.849 (0.549-1.314)	0.462
rs1169288				
AA	168 (35.7%)	67 (30.6%)	1.000 (reference)	
AC	221 (46.9%)	119 (54.3%)	1.108 (0.724-1.697)	0.637
CC	82 (17.4%)	33 (15.1%)	0.847 (0.474-1.511)	0.573
AC+CC	303 (64.3%)	152 (69.4%)	1.036 (0.691-1.552)	0.865
rs1169286				
TT	136 (28.9%)	58 (26.5%)	1.000 (reference)	
TC	224 (47.6%)	116 (53.0%)	1.017 (0.650-1.590)	0.943
CC	111 (23.5%)	45 (20.5%)	0.865 (0.501-1.495)	0.603
TC+CC	335 (71.1%)	161 (73.5%)	0.968 (0.635-1.475)	0.878

aORs (95% CIs) were estimated using multiple logistic regression models after pathologic Gleason scores, clinical T stages, pathologic T and N stages, seminal vesicle invasion, perineural invasion, lymphovascular invasion, and D'Amico risk classification were controlled for.

**Table 3:** ORs (95% CIs) of the clinical status and *HNF1A* rs735396 and rs2464196 genotypic frequencies in included patients.

Variable	rs735396				rs2464196			
	TT (*n* = 173)	TC+CC (*n* = 517)	OR (95% CI)	*p*	GG (*n* = 177)	GA+AA (*n* = 513)	OR (95% CI)	*p*
Pathologic Gleason grade group								
1 or 2	119 (68.8%)	294 (56.9%)	**1.000**	0.006*	121 (68.4%)	292 (56.9%)	**1.000**	0.007*
3, 4, or 5	54 (31.2%)	223 (43.1%)	**1.672 (1.160-2.409)**		56 (31.6%)	221 (43.1%)	**1.635 (1.139-2.348)**	
Clinical T stage								
1 or 2	152 (87.9%)	441 (85.3%)	1.000	0.401	156 (88.1%)	437 (85.2%)	1.000	0.330
3 or 4	21 (12.1%)	76 (14.7%)	1.247 (0.744-2.092)		21 (11.9%)	76 (14.8%)	1.292 (0.771-2.166)	
Pathologic T stage								
2	96 (55.5%)	267 (51.6%)	1.000	0.380	97 (54.8%)	266 (51.9%)	1.000	0.498
3 or 4	77 (44.5%)	250 (48.4%)	1.167 (0.826-1.650)		80 (45.2%)	247 (48.1%)	1.126 (0.799-1.586)	
Pathologic N stage								
N0	164 (94.8%)	467 (90.3%)	1.000	0.069	168 (94.9%)	463 (90.3%)	1.000	0.056
N1	9 (5.2%)	50 (9.7%)	1.951 (0.939-4.055)		9 (5.1%)	50 (9.7%)	2.016 (0.970-4.189)	
Seminal vesicle invasion								
No	141 (81.5%)	402 (77.8%)	1.000	0.298	144 (81.4%)	399 (77.8%)	1.000	0.316
Yes	32 (18.5%)	115 (22.2%)	1.260 (0.815-1.950)		33 (18.6%)	114 (22.2%)	1.247 (0.810-1.920)	
Perineural invasion								
No	50 (28.9%)	131 (25.3%)	1.000	0.356	51 (28.8%)	130 (25.3%)	1.000	0.365
Yes	123 (71.1%)	386 (74.7%)	1.198 (0.816-1.758)		126 (71.2%)	383 (74.7%)	1.192 (0.814-1.746)	
Lymphovascular invasion								
No	152 (87.9%)	427 (82.6%)	1.000	0.102	155 (87.6%)	424 (82.7%)	1.000	0.125
Yes	21 (12.1%)	90 (17.4%)	1.526 (0.916-2.540)		22 (12.4%)	89 (17.3%)	1.479 (0.896-2.442)	
D'Amico risk classification								
Low or intermediate risk	86 (49.7%)	255 (49.3%)	1.000	0.930	88 (49.7%)	253 (49.3%)	1.000	0.927
High risk	87 (50.3%)	262 (50.7%)	1.016 (0.720-1.433)		89 (50.3%)	260 (50.7%)	1.016 (0.722-1.430)	

ORs with their 95% CIs were estimated using logistic regression models. * *p* < 0.05 as statistically significant.

**Table 4:** ORs (95% CIs) of the clinical status and *HNF1A* rs1169288 and rs1169286 genotypic frequencies in included patients.

Variable	rs1169288				rs1169286			
	AA (*n* = 235)	AC+CC (*n* = 455)	OR (95% CI)	*p*	TT (*n* = 194)	TC+CC (*n* = 496)	OR (95% CI)	*p*
Pathologic Gleason grade group								
1 or 2	153 (65.1%)	260 (57.1%)	1.000	0.043*	125 (64.4%)	288 (58.1%)	1.000	0.125
3, 4, or 5	82 (34.9%)	195 (42.9%)	**1.399 (1.010-1.939)**		69 (35.6%)	208 (41.9%)	1.308 (0.928-1.845)	
Clinical T stage								
1 or 2	208 (88.5%)	385 (84.6%)	1.000	0.163	169 (87.1%)	424 (85.5%)	1.000	0.580
3 or 4	27 (11.5%)	70 (15.4%)	1.401 (0.871-2.252)		25 (12.9%)	72 (14.5%)	1.148 (0.704-1.871)	
Pathologic T stage								
2	129 (54.9%)	234 (51.4%)	1.000	0.388	105 (54.1%)	258 (52.0%)	1.000	0.618
3 or 4	106 (45.1%)	221 (48.6%)	1.149 (0.838-1.576)		89 (45.9%)	238 (48.0%)	1.088 (0.780-1.518)	
Pathologic N stage								
N0	233 (94.9%)	408 (89.7%)	1.000	0.020*	183 (94.3%)	448 (90.3%)	1.000	0.091
N1	12 (5.1%)	47 (10.3%)	2.141 (1.112-4.120)		11 (5.7%)	48 (9.7%)	1.782 (0.905-3.509)	
Seminal vesicle invasion								
No	193 (82.1%)	350 (76.9%)	1.000	0.114	157 (80.9%)	386 (77.8%)	1.000	0.370
Yes	42 (17.9%)	105 (23.1%)	1.379 (0.925-2.054)		37 (19.1%)	110 (22.2%)	1.209 (0.798-1.833)	
Perineural invasion								
No	63 (26.8%)	118 (25.9%)	1.000	0.805	50 (25.8%)	131 (26.4%)	1.000	0.864
Yes	172 (73.2%)	337 (74.1%)	1.046 (0.732-1.494)		144 (74.2%)	365 (73.6%)	0.967 (0.663-1.413)	
Lymphovascular invasion								
No	204 (86.8%)	375 (82.4%)	1.000	0.137	167 (86.1%)	412 (83.1%)	1.000	0.332
Yes	31 (13.2%)	80 (17.6%)	1.404 (0.897-2.198)		27 (13.9%)	84 (16.9%)	1.261 (0.789-2.016)	
D'Amico classification								
Low or intermediate risk	128 (54.5%)	213 (46.8%)	1.000	0.057	95 (49.0%)	246 (49.6%)	1.000	0.882
High risk	107 (45.5%)	242 (53.2%)	1.359 (0.991-1.864)		99 (51.0%)	250 (50.4%)	0.975 (0.700-1.359)	

ORs with their 95% CIs were estimated using logistic regression models. * *p* < 0.05 as statistically significant.

**Table 5:** ORs (95% CIs) of the clinical status and *HNF1A* rs735396 genotypic frequencies in included patients with or without BCR.

Variable	No BCR (n = 471)				BCR (n = 219)			
	TT (*n* = 122)	TC+CC (*n* = 349)	OR (95% CI)	*p*	TT (*n* = 51)	TC+CC (*n* = 168)	OR (95% CI)	*p*
Pathologic Gleason grade group								
1 or 2	94 (77.0%)	249 (71.3%)	1.000	0.223	25 (49.0%)	45 (26.8%)	**1.000**	0.003*
3, 4, or 5	28 (23.0%)	100 (28.7%)	1.348 (0.833-2.182)		26 (51.0%)	123 (73.2%)	**2.628 (1.377-5.017)**	
Clinical T stage								
1 or 2	113 (92.6%)	316 (90.5%)	1.000	0.488	39 (76.5%)	125 (74.4%)	1.000	0.766
3 or 4	9 (7.4%)	33 (9.5%)	1.311 (0.608-2.825)		12 (23.5%)	43 (25.6%)	1.118 (0.537-2.329)	
Pathologic T stage								
2	79 (64.8%)	232 (66.5%)	1.000	0.730	17 (33.3%)	35 (20.8%)	1.000	0.066
3 or 4	43 (35.2%)	117 (33.5%)	0.927 (0.601-1.428)		34 (66.7%)	133 (79.2%)	1.900 (0.952-3.792)	
Pathologic N stage								
N0	121 (99.2%)	338 (96.8%)	1.000	0.159	43 (84.3%)	129 (76.8%)	1.000	0.251
N1	1 (0.8%)	11 (3.2%)	3.938 (0.503-30.823)		8 (15.7%)	39 (23.2%)	1.625 (0.705-3.747)	
Seminal vesicle invasion								
No	110 (90.2%)	316 (90.5%)	1.000	0.902	31 (60.8%)	86 (51.2%)	1.000	0.229
Yes	12 (9.8%)	33 (9.5%)	0.957 (0.478-1.919)		20 (39.2%)	82 (48.8%)	1.478 (0.781-2.798)	
Perineural invasion								
No	44 (36.1%)	119 (34.1%)	1.000	0.694	6 (11.8%)	12 (7.1%)	1.000	0.293
Yes	78 (63.9%)	230 (65.9%)	1.090 (0.709-1.677)		45 (88.2%)	156 (92.9%)	1.733 (0.616-4.877)	
Lymphovascular invasion								
No	114 (93.4%)	323 (92.6%)	1.000	0.743	38 (74.5%)	104 (61.9%)	1.000	0.099
Yes	8 (6.6%)	26 (7.4%)	1.147 (0.505-2.606)		13 (25.5%)	64 (38.1%)	1.799 (0.891-3.632)	
D'Amico classification								
Low or intermediate risk	68 (55.7%)	198 (56.7%)	1.000	0.849	18 (35.3%)	57 (33.9%)	1.000	0.857
High risk	54 (44.3%)	151 (43.3%)	0.960 (0.634-1.455)		33 (64.7%)	111 (66.1%)	1.062 (0.551-2.049)	

ORs with their 95% CIs were estimated using logistic regression models. * *p* < 0.05 as statistically significant.

**Table 6:** ORs (95% CIs) of clinical status and *HNF1A* rs2464196 genotypic frequencies in included patients with or without BCR.

Variable	No BCR (n = 471)				BCR (n = 219)			
	GG (*n* = 124)	GA+AA (*n* = 347)	OR (95% CI)	*p*	GG (*n* = 53)	GA+AA (*n* = 166)	OR (95% CI)	*p*
Pathologic Gleason grade group								
1 or 2	96 (77.4%)	247 (71.2%)	1.000	0.180	25 (47.2%)	45 (27.1%)	**1.000**	0.006*
3, 4, or 5	28 (22.6%)	100 (28.8%)	1.388 (0.858-2.245)		28 (52.8%)	121 (72.9%)	**2.401 (1.268-4.547)**	
Clinical T stage								
1 or 2	115 (92.7%)	314 (90.5%)	1.000	0.450	41 (77.4%)	123 (74.1%)	1.000	0.634
3 or 4	9 (7.3%)	33 (9.5%)	1.343 (0.623-2.893)		12 (22.6%)	43 (25.9%)	1.194 (0.575-2.481)	
Pathologic T stage								
2	80 (64.5%)	231 (66.6%)	1.000	0.678	17 (32.1%)	35 (21.1%)	1.000	0.102
3 or 4	44 (35.5%)	116 (33.4%)	0.913 (0.594-1.404)		36 (67.9%)	131 (78.9%)	1.767 (0.889-3.513)	
Pathologic N stage								
N0	123 (99.2%)	336 (96.8%)	1.000	0.152	45 (84.9%)	127 (76.5%)	1.000	0.195
N1	1 (0.8%)	11 (3.2%)	4.027 (0.515-31.515)		8 (15.1%)	39 (23.5%)	1.727 (0.751-3.974)	
Seminal vesicle invasion								
No	112 (90.3%)	314 (90.5%)	1.000	0.957	32 (60.4%)	85 (51.2%)	1.000	0.244
Yes	12 (9.7%)	33 (9.5%)	0.981 (0.490-1.965)		21 (39.6%)	81 (48.8%)	1.452 (0.774-2.724)	
Perineural invasion								
No	45 (36.3%)	118 (34.0%)	1.000	0.646	6 (11.3%)	12 (7.2%)	1.000	0.345
Yes	79 (63.7%)	229 (66.0%)	1.105 (0.720-1.696)		47 (88.7%)	154 (92.8%)	1.638 (0.583-4.603)	
Lymphovascular invasion								
No	116 (93.5%)	321 (92.5%)	1.000	0.701	39 (73.6%)	103 (62.0%)	1.000	0.126
Yes	8 (6.5%)	26 (7.5%)	1.174 (0.517-2.668)		14 (26.4%)	63 (38.0%)	1.704 (0.858-3.385)	
D'Amico classification								
Low or intermediate risk	69 (55.6%)	197 (56.8%)	1.000	0.828	19 (35.8%)	56 (33.7%)	1.000	0.778
High risk	55 (44.4%)	150 (43.2%)	0.955 (0.632-1.444)		34 (64.2%)	110 (66.3%)	1.098 (0.575-2.096)	

ORs with their 95% CIs were estimated using logistic regression models. * *p* < 0.05 as statistically significant.
